# Aptamer-conjugated multi-walled carbon nanotubes as a new targeted ultrasound contrast agent for the diagnosis of prostate cancer

**DOI:** 10.1007/s11051-018-4407-z

**Published:** 2018-11-12

**Authors:** Fenfen Gu, Chuling Hu, Qingming Xia, Chunai Gong, Shen Gao, Zhongjian Chen

**Affiliations:** 1 Department of Pharmacy, Xinhua Hospital, Shanghai Jiaotong University School of Medicine, No. 1665 Kongjiang Road, Yangpu District, Shanghai, 200092 China; 2 Department of Pharmacy, Changhai Hospital, Second Military Medical University, No. 168 Changhai Road, Yangpu District, Shanghai, 200092 China; 3 Department of Pharmacy, Jiaxing Maternity and Child Health Care Hospital, No. 2468, Central ring road, Jiaxing, 314000 China; 4 Department of Pharmacy, Shanghai Dermatology Hospital, No. 200, Wuyi Road, Changning District, Shanghai, 200050 China

**Keywords:** Multi-walled carbon nanotube, Prostate cancer, Aptamer, Ultrasound, Visualization contrast agent, Nanomedicine

## Abstract

Early diagnosis is primarily important for the therapeutic and prognostic outcomes of malignancies including prostate cancer (PCa). However, the visuality and veracity of ultrasound imaging for the diagnosis and prognostic prediction of PCa remains poor at present. In this study, we developed a new nanoultrasound contrast agent by modifying multi-walled carbon nanotubes (MWCNTs) with polyethylene glycol (PEG) and anti-PSMA aptamer. The result showed that the modified MWCNTs offered better visuality and veracity and were able to target PCa cells more effectively as compared with the traditional contrast agent. The zeta potential was about − 38 mv. The length of this contrast agent was about 400 nm and the diameter of it was about 30 nm. The zeta potential, TEM, and FT-IR all proved the successful preparation of the agent. The vitro cytological study revealed good cell uptake and biocompatibility of the new contrast agent. The minimum detection concentration in vitro is 10 μg/ml. The earliest stage of the detection was under the parameters of frequency = 6.0 MHz and medical index = 0.06. Both in vitro and in vivo ultrasound imaging demonstrated that the new nanoultrasound contrast agent had a good development effect, distribution, and metabolism, and may prove to be a good targeted ultrasound contrast agent, especially for PCa.

## Introduction

Prostate cancer (PCa) is one of the most frequently diagnosed cancers all over the world and the leading cause of cancer-related death in males in many countries. About 1.8 million new PCa cases were reported in 2016 (Siegel et al. 2016). Radical resection based on early diagnosis is the key to survival of cancer patients (Helmstaedter and Riemann 2008). However, the early diagnosis and resection rate of PCa is only about 10–20% at present (Bilimoria et al. 2008). Prostate biopsy performed by real-time image-guided transrectal ultrasound (TRUS) is the golden criterion for PCa diagnosis (Baur et al. 2017). Unfortunately, systematic biopsies usually have to be performed due to insufficient visualization of PCa on brightness-mode TRUS. This poor visuality results in the poor detection rate and incorrect diagnosis of insignificant cancers (Shakir et al. 2017; Welch and Albertsen 2009; Ahmed et al. 2017; Ukimura et al. 2013; Washington et al. 2012).

With advances in material science and technology, ultrasonic contrast agents have been designed to meet different requirements by using polymers, capsids, and liposomes (Paefgen et al. 2015). However, most of these ultrasound contrast agents are unstable under insonification pressure and distribute widely with poor circulating acoustic contrast properties (Hahn et al. 2011; Kiessling et al. 2012). Recently, inorganic ultrasonic imaging materials have aroused increasing attention and interest due to their regulator particle size, good biocompatibility, and superior stability compared with the conventional contrast agents (Chen et al. 2012. Of these inorganic materials, multi-walled carbon nanotubes (MWCNTs) have been reported as a more promising ultrasound contrast material due to their unique structure and properties (Delogu et al. 2012; Wu et al. 2014). As we all know, tumor has enhanced permeability and retention effect (EPR). Nanoparticles can passively target tumors through the EPR effect of tumors. So MWCNTs can target to the tumor passively to diagnosis tumor. When MWCNTs were modified with ligand, it can target to the specific tumor, which improved the accuracy and sensitivity of diagnosis.

Prostate-specific membrane antigen (PSAM) is a specific membrane protein expressed by PCa cells and its expression is positively correlated with PCa aggression. Studies have shown that PSMA is highly expressed in metastatic PCa androgen-independent prostate cancer (AIPC) (Ristau et al. 2014). Currently, many anti-PSAM ligands including monoclonal antibodies, programmed antibodies, and small molecular substances have been reported (Wang et al. 2013; Xiao et al. 2015). Nucleic acid aptamers are some nucleotide fragments that can specifically combine proteins or other small molecules screened by vitro SELEX screening technology. They were found to have high affinity and good specificity to target ligands (Xiao et al. 2016). As RNA nucleic acid is composed of nucleotide and has many structural alterations and strong specificity, it is often used in molecular imaging and gene therapy (Pai and Ellington 2009; McNamara et al. 2006; Baek et al. 2014; Chu et al. 2006; Mathew et al. 2015). In addition, nucleic acid as a targeted probe can avoid immunogenicity, and its high purity makes it safer than other ligands (Dougherty et al. 2015; Alibolandi et al. 2015; Lee et al. 2011). Early aptamers A10 and A9 were selected from the synthetic RNA aptamer sequences and effectively connected to the PSMA (Rockey et al. 2011; Wu et al. 2011). However, they are composed of 79 nucleotides. This high molecular weight severely limits its targeting ability. The A10-3.2 aptamer used in this study was composed of 37 nucleotides with a greatly reduced molecular weight but without affecting its targeting ability (Rockey et al. 2011; Wu et al. 2011).

Based on the above research background and foundation, we developed a new nanoultrasound contrast agent by modifying MWCNTs with polyethylene glycol (PEG) and PSMA targeted aptamer A10-3.2 and evaluated its cytological characteristics, targeting ability, safety, developing ability, metabolism, and distribution both in vivo and in vitro. The results showed that the new ultrasound nanocontrast agent had a good targeting ability and was able to detect early-stage PCa.

## Materials and methods

### Materials

Materials used in this study were MWCNTs (length 400 nm, diameter 15 nm) (Cheap Tubes Inc., Brattleboro, VT, USA); NH2-PEG-COOH (polyethylene glycol modified by amino and carboxyl) (MW: 2000) (BO Biological Technology Co., Ltd., Jiaxing, China); EDC(1-(3-Dimethylaminopropyl)-3-ethylcarbodiimide hydrochloride) and NHS (N-Hydroxysuccinimide;1-hydroxypyrrolidine-2,5-dione) (Aladdin Biological Technology Co., Ltd., Shanghai, China); Coumarin 6 (Thermo Fisher Scientific, MA,USA); 4′,6-diamidino-2-phenylindole (DAPI) (Sigma-Aldrich, St. Louis, MO,USA); Dulbecco’s modified Eagle’s medium (DMEM), fetal bovine serum (FBS), and penicillin-streptomycin solution (5 kU/mL) (Life Technologies, Grand Island, USA); Cell Counting Kit-8 (CCK-8) (Dojindo Molecular Technologies Inc., Nanjing, China); and anti-PSMA aptamer (sequence 5′-GGGAGGACGAUGCGGAUCAGCCAUGUUUACGUCACUCCU-3′ with 5′ modification of amino group) (RiboBio Co., Ltd., Guangzhou, China).

All other reagents were of analytical grade. All animal experiments were performed in accordance with the ethics and regulations of animal experiments of the Second Military Medical University (Shanghai, China).

### Cell lines and culture

PC-3 cells overexpressing PSMA (SBO Medical Biotechnology, Shanghai, China) were cultured in RPMI 1640 with 10% FBS and 1% penicillin-streptomycin, and incubated under 5% CO2 atmosphere at 37 °C.

### Animals

Male BALB/c nude mice (4 weeks) were supplied by the Department of Experimental Animals of the Second Military Medical University. The animal experimental procedures were in agreement with the guidelines of the Institutional Animal Care and Use Committee (IACUC) of Shanghai Institute of Materia Medica of the Chinese Academy of Sciences (Shanghai, China).

### Synthesis of CNT-PEG-Ap

To stabilize MWCNTs in a solution, PEG-coated MWCNTs were prepared, given the highly hydrophobic surface of MWCNTs.

Pristine nanotubes were initially oxidized to obtain MWCNT-COOH. Briefly, a mixture of concentrated nitric acid and sulfuric acid (1:3, *v*/*v*, 80 ml) was added to MWCNTs (2 g), put in an ultrasonic bath (40 kHz) at 40 °C for 7 h, and stirred for additional 24 h at room temperature to complete the reaction. After that, 500 ml pure water was added to the mixture. The MWCNTs functionalized with carboxylic acid group were separated from the solution by centrifugation, washed with water, and dried by lyophilization.

To prepare CNT-PEG, MWCNT-COOH (30 mg) was dispersed in 150 ml methanol, and then 0.3 g N-(3-dimethylaminopropyl)-N′-ethylcarbodiimide hydrochloride (EDC) and 0.2 g N-hydroxysuccinimide (NHS) were added to the solution to obtain MWCNT-COOH. Three hours after stirring at room temperature, the activated MWCNT-COOH was separated from the solution by centrifugation, rinsed with methanol repeatedly, and then dried by lyophilization. After that, 10 mg NH2-PEG-COOH was added to the activated MWCNT-COOH solution (containing 20 mg MWCNT-COOH and 50 ml methanol), followed by another 24-h stirring. The resulting CNT-PEG-COOH was separated, washed, and dried by lyophilization.

For conjugation of the aptamer, MWCNT-PEG-COOH (10 mg) was dispersed in 10 mL phosphate buffered saline (PBS), followed by addition of 0.15 g EDC and 0.1 g NHS to the solution to activate MWCNT-PEG-COOH for 3 h. Then 50 nM Anti-PSMA aptamer with 5′ modification of amino group was added to the solution and stirred for 24 h at room temperature. Then, MWCNT-PEG-Ap was separated by centrifugation, rinsed with pure water repeatedly, and dried by lyophilization.

### Characterization of the complex

Fourier transform infrared spectroscopy (FT-IR) (Agilent Cary 670, Australia) was used to determine the synthesized polymers. The particle size and zeta potential of MWCNT-PEG-Ap were determined using dynamic light scattering (Zetasizer Nano ZS90, Malvern Instruments, USA). The morphology of MWCNT-PEG-Ap was observed by transmission electron microscopy (TEM) (Hitachi, Tokyo, Japan) at an acceleration voltage of 75 kV.

### Flow cytometry

MWCNT-PEG-Ap was labeled with Coumarin 6. Briefly, MWCNT-PEG-Ap was incubated with Coumarin 6 in methanol for 6 h under dark conditions. The Coumarin 6-loaded MWCNT-PEG-Ap (MWCNT-PEG-Ap/Coumarin 6) was collected by centrifugation and washed 3 times to remove the excess Coumarin 6. PC-3 cells overexpressing PSMA were cultured in a 12-well plate at 2 × 10^5^ cells per well and incubated for 24 h for in vitro study. Then the culture medium was replaced, and different concentrations of MWCNT-PEG-Ap/Coumarin 6 were added. After 3-h incubation, cells were washed, collected, and analyzed on a FACScan flow cytometer (Becton Dickinson, San Jose, CA, USA).

### Fluorescent imaging in cells

PC-3 cells were plated in glass-base dishes at a density of 1.0 × 10^5^ per dish and incubated for 24 h. Then, the medium was replaced with 0.5 ml fresh medium containing MWCNT-PEG-Ap/Coumarin 6 or an equivalent concentration of free Coumarin 6 (100 ng/mL). After 3-h incubation, cells were washed three times with PBS and fixed with 4% paraformaldehyde. After staining with DAPI, the cells were washed and observed under a confocal laser scanning microscope (Olympus, Japan).

### In vitro cytotoxicity

The cytotoxicity of MWCNT-PEG-Ap on PC-3 cells was performed using CCK-8 assay. Briefly, PC-3 cells (5 × 10^3^ cell/well) were seeded in 96-well plates and incubated for 24 h. Then the culture medium was replaced by fresh medium containing different concentrations of MWCNT-PEG-Ap for 24 h, followed by addition of 10 μl CCK-8 solution to each well. After 2-h incubation, the absorbance was measured with a microplate reader (Thermo, IL, USA) at 450 nm. The cell viability was expressed as a percentage relative to the absorbance of the untreated samples.

### In vitro US imaging analysis

Ultrasound images in vitro with PBS control, MWCNTs, and MWCNT-PEG-Ap were carried out on the Mylab 90 scanner (Esaote Medical Systems, Genova, Italy) under the parameters of frequency = 6.0 MHz and medical index = 0.06. Typically, the Eppendorf tube (2 mL) was filled with PBS solution of a constant concentration of samples, and then the tube was immerged in a pure water tank. The transducer was coated with the US gel to avoid air background. All images were recorded as digital files for subsequent playback and analysis.

### In vivo US imaging

In vivo US imaging was performed in the BALB/c xenograft nude mice models. The models were generated by subcutaneous injection of 1 × 10^7^ PC-3 cells. When the diameter of the tumor reached 0.8–1.0 cm, the mice were randomly divided into three groups. All the mice were anesthetized by intraperitoneal injection of 2% pentobarbital and then intravenously injected with 200 μl (a) PBS, (b) MWCNT-PEG, or (c) MWCNT-PEG-Ap (10 mg/kg per injection) respectively via the tail vein. Images of the tumor, heart, and kidney were taken at 1, 8, and 24 h after injection.

### Statistical analysis

Line charts were made by GraphPad Prism 5.0. Statistical analysis was performed using SPSS 22.0. All data were compared and analyzed using the paired-sample Student’s *t* test. *P* < 0.05 indicated statistical significance.

## Results and discussion

### Characterization of MWCNT-PEG-Ap

**Fig. 1:**
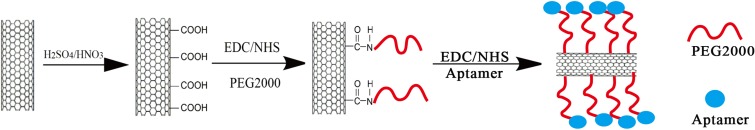
Synthesis of MWCNT-PEG-Ap

The use of conventional MWCNTs as an ultrasound contrast enhancement agent is largely limited by their poor water solubility. The MWCNTs were initially oxidized and shortened to introduce carboxylic acid groups onto the side walls of MWCNTs. Knowing that PEG is widely used as a hydrophilic substance to improve water solubility of drugs and escape the removal by the reticuloendothelial system (RES). So we introduced PEG to MWCNTs to increase their stability and water solubility. Anti-PSMA aptamer with 5′ modification of amino group was linked with the MWCNT-PEG to target PCa cells. The schematic diagram is shown in Fig. 1.

**Fig. 2:**
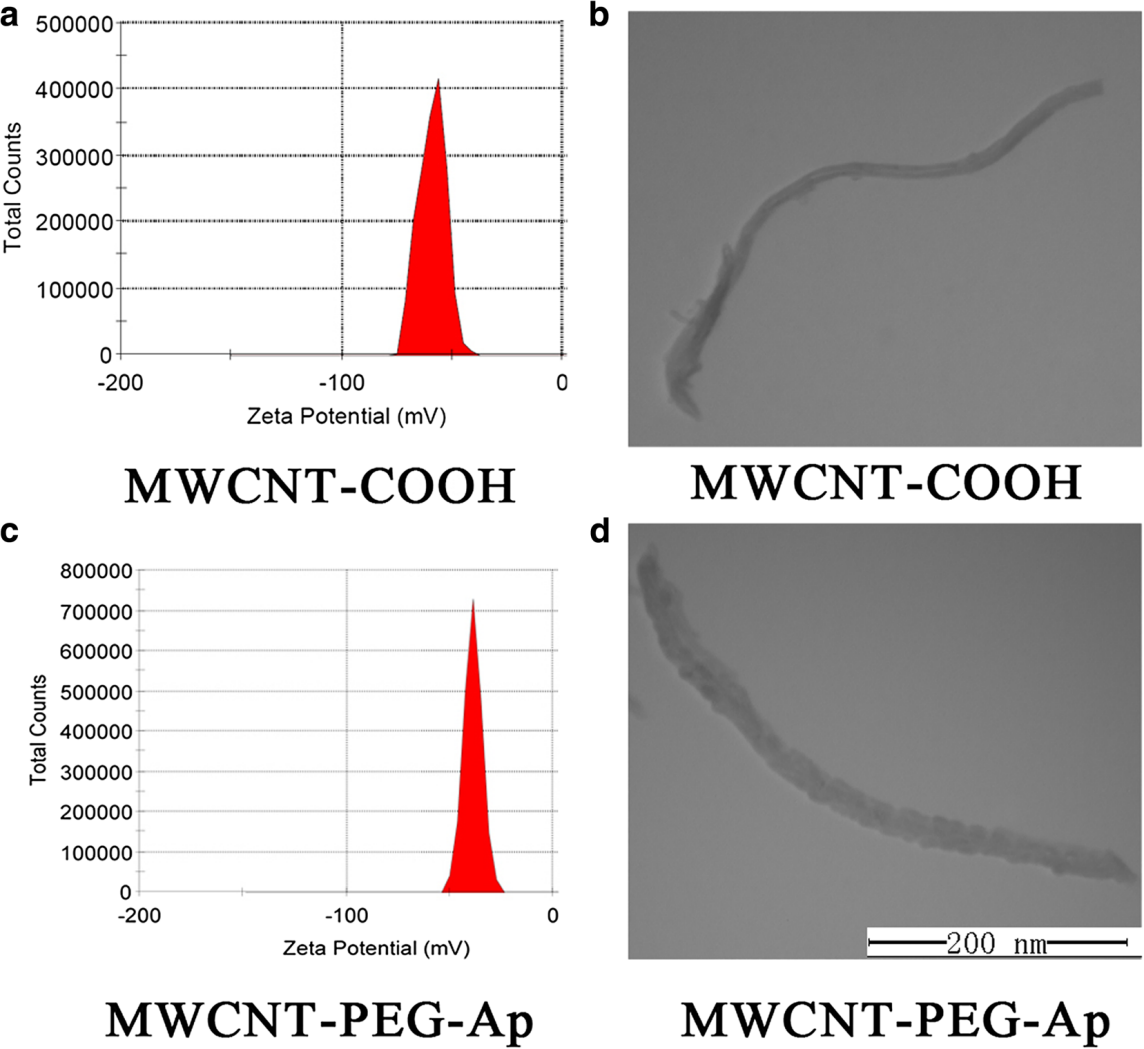
Zeta potential and TEM of the ultrasound contrast agent. **a**, **c** Zeta potential of the ultrasound contrast agent. **b**, **d** TEM of the ultrasound contrast agent

**Fig. 3:**
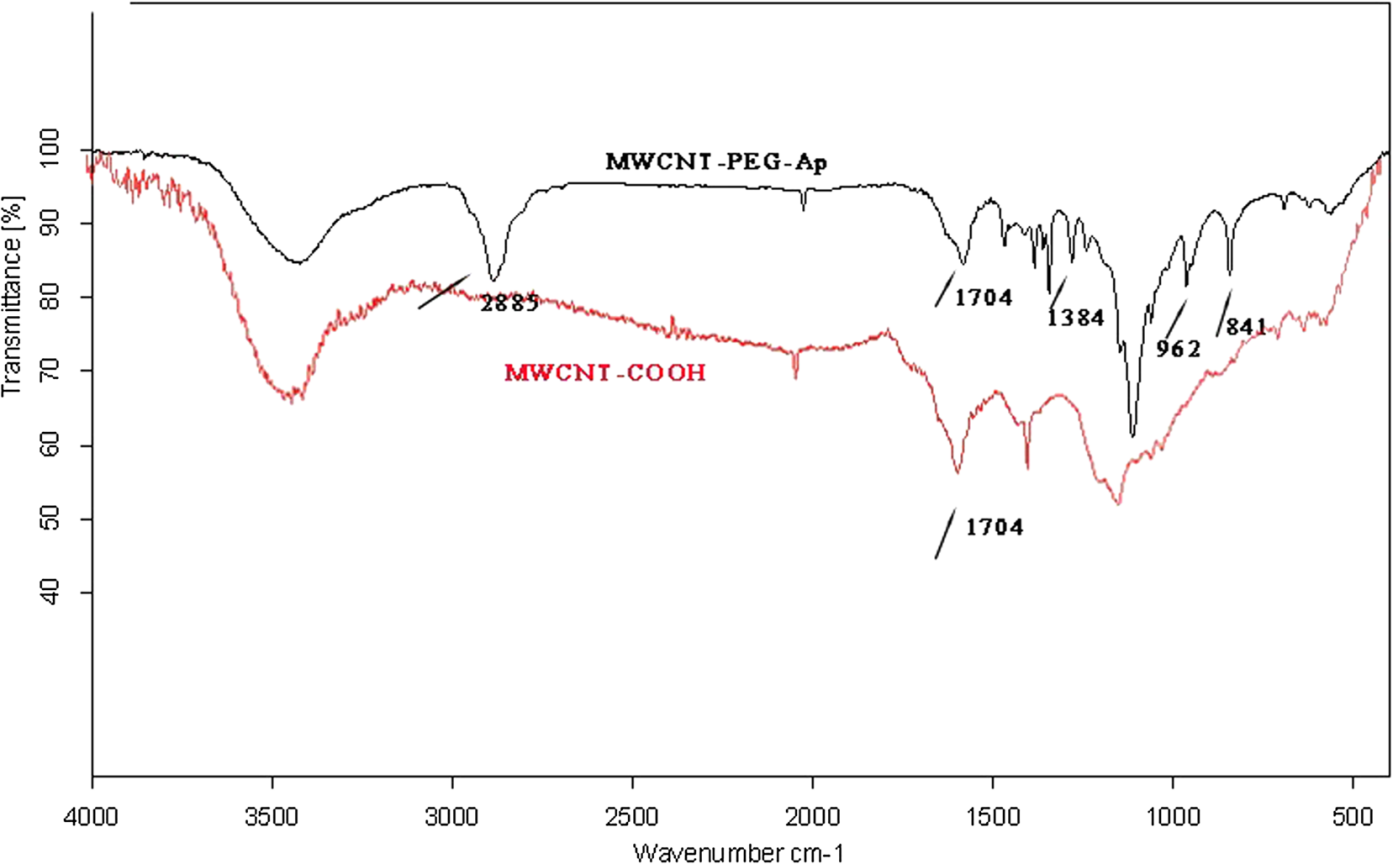
FT-IR of the ultrasound contrast agent

Size and zeta potential are common characterization of nanoparticles. As shown in Fig. 2a, c, the zeta potential of MWCNT-PEG was − 50 mv while the zeta potential of MWCNT-PEG-Ap was − 38.8 mv. TEM showed that the new ultrasound contrast agent was strip-shaped with good dispersion and the aptamer connected the surface of the nanotube successfully (Fig. 2b, d). From the ruler, we can know that the length of this contrast agent was about 400 nm and the diameter of the aptamer unconnected MWCNTs was about 15 nm. When the aptamer was successfully linked, the particle diameter became 30 nm and the surface became rough. All these indicate that the aptamer is successfully connected. In addition, FT-IR showed that MWCNT-PEG-Ap was synthesized successfully (Fig. 3). The main absorption peak of MWCNT-COOH appeared at 1704 cm^−1^, attributed to the carbonyl group of MWCNT-COOH (Hu et al. 2005). The new absorption peak at 2885 cm^−1^was ascribed to –CH2-symmrtrical stretching vibration and the new peak at 962 cm^−1^ belonged to –C–O–C– in-plane deformation vibration (Chen et al. 2001). In addition, the peak at 841 resulting from C–N flexural vibration and the degree of absorption at 1704 cm^−1^ were significantly decreased, indicating the successful combination of MWCNT-COOH with PEG200. All these results indicated the successful synthesis of MWCNT-PEG-Ap.

### Cellular uptake

**Fig. 4:**
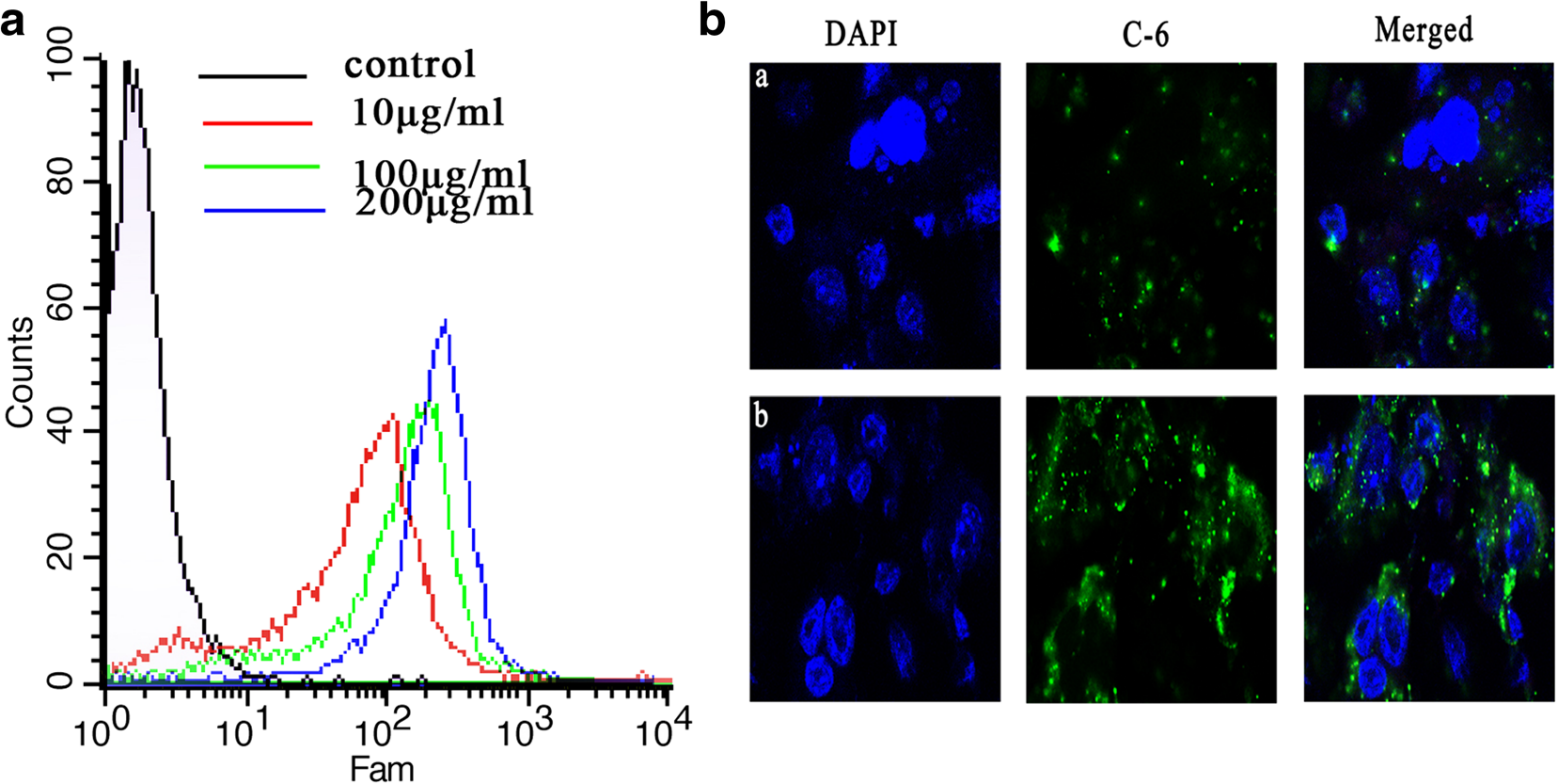
The cellular uptake of MWCNT-PEG-Ap. **a** Flow cytometry of cellular uptake of different concentrations. **b** CLSM of cellular uptake: (a) free Coumarin 6; (b) MWCNT-PEG-Ap/Coumarin 6

Effective cellular uptake is essential to drug delivery. Flow cytometry and CLSM were used to evaluate the cellular uptake of MWCNT-PEG-Ap, and the results are shown in Fig. 4. We investigated the uptake of a series of concentrations to PC3 cells. As shown in Fig. 4a, the uptake rate was nearly 100% at the concentration of 200 μg/mL by MWCNT-PEG-Ap, and the mean fluorescence intensity was almost sixfold the uptake rate of the concentration at 10 μg/mL. Figure 4b shows the CLSM of cellular uptake at the concentration of 100 μg/ml. The fluorescence intensity in MWCNT-PEG-Ap/Coumarin 6 group was significantly higher than that in free Coumarin 6 group, indicating that MWCNT-PEG-Ap had good cellular uptake and mostly localized in the cytoplasm.

### Cytotoxicity assay

**Fig. 5:**
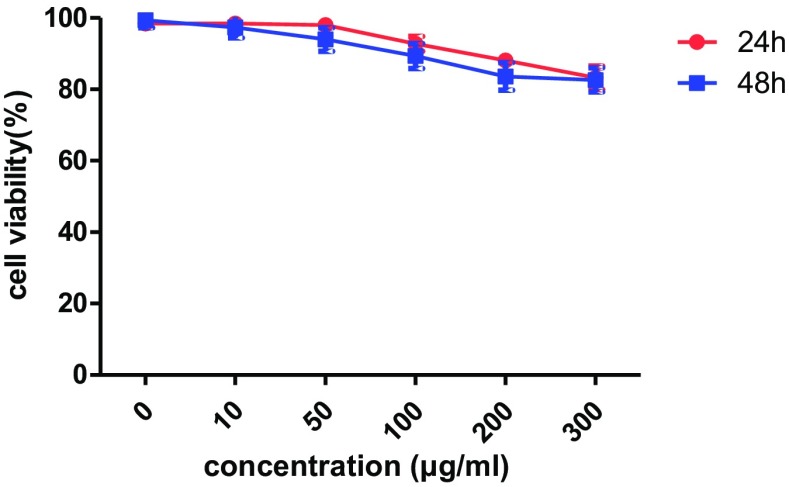
Cytotoxicity of MWCNT-PEG-Ap

Knowing that safety, low toxicity, and good biocompatibility are essential for any nanocontrast agent, we evaluated the cytotoxicity of MWCNT-PEG-Ap by CCK-8 assay. The results are shown in Fig. 5.

As shown in Fig. 5, the viability of PC3 cells remained almost unchanged when the concentration went up. When the concentration was increased to 300 μg/ml, the cell viability was about 80% after 24-h treatment, and with the lapse of time, the cell survival rate did not change significantly. This may be due to the fact that before the uptake reaches saturation, with the increase of concentration or time, nanomaterials enter the cell more, thus reducing cell viability. When the uptake reached saturation, the amount of nanomaterials kept constant in cells, so the cell viability remained almost unchanged even with increasing concentration or prolonging time. These all indicated that the nanomaterial had good biocompatibility and was very safe.

### In vitro US imaging

**Fig. 6:**
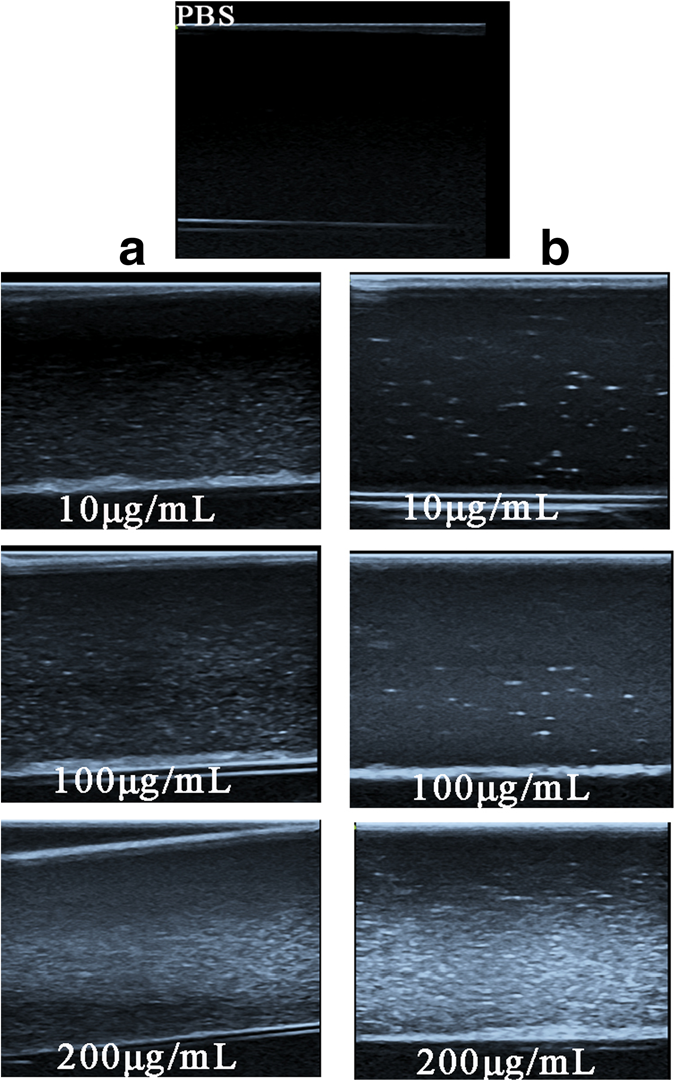
In vitro imaging of MWCNT-PEG2000-Ap and MWCNT-PEG2000 at different concentrations. (A) MWCNT-PEG2000 group. (B) MWCNT-PEG2000-Ap group

The vitro images of different groups were observed and the results are shown as Fig. 6. Two-dimensional (2D) gray-scale US and contrast-enhanced US (CEUS) were used as imaging models (He Hu et al. 2011). There was almost no image shown in PBS control group. However, images were clearly shown in MWCNT-PEG2000 and MWCNT-PEG-Ap groups in a concentration-dependent manner. The minimum detection concentration in vitro is 10 μg/ml. The earliest stage of the detection was under the parameters of frequency = 6.0 MHz and medical index = 0.06.With the concentration increasing, the images of the ultrasound were progressively enhanced. In addition, there was no significant difference in imaging between MWCNT-PEG2000 and MWCNT-PEG2000-Ap in vitro. The images emerged clearly at the concentration of 10 μg/ml, which is much lower than the lowest concentration of hollow silica microspheres used under similar experimental conditions (He Hu et al. 2011; Yu et al. 2012). These results indicated that MWCNT-PEG2000-Ap was an effective ultrasound contrast agent.

### In vivo US imaging

**Fig. 7:**
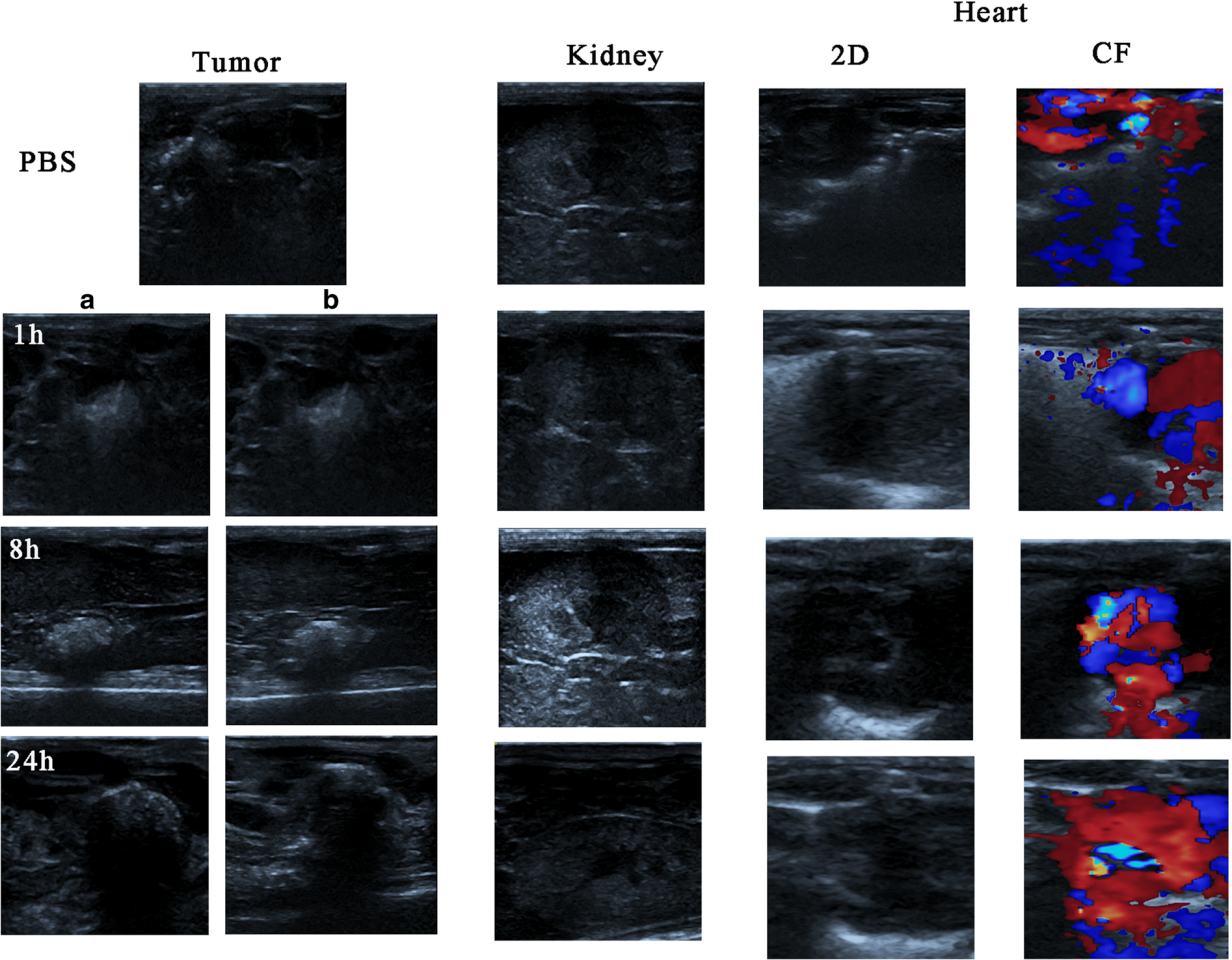
In vivo US images. (A) MWCNT-PEG2000 group. (B) MWCNT-PEG2000-Ap group. The kidney and heart US images were carried by the group B

The in vivo enhancement effect was assessed in the transplanted tumor model and the results are shown in Fig. 7.

The tumor, heart, and kidney were observed and recorded at different time points after injection of the contrast agent. The images were barely visible in the control group. However, the contrast enhancement of the tumor site and kidney was observable in MWCNT-PEG2000 group and MWCNT-PEG2000-Ap group and was the clearest 8 h after injection. The US signal in MWCNT-PEG2000-Ap group was stronger than that in MWCNT-PEG2000 group, while no US signal was observed in the heart. These results indicated that this contrast agent had a good enhancement effect in vivo. As the time prolonged, the enhancement signal increased in the first 8 h and then decreased gradually. This may because that since the contrast agent is injected, the contrast agent gradually distributes from the caudal vein to the tissue. The results showed that the complete distribution time of the contrast medium was about 8 h, followed by metabolic excretion. We also investigated the distribution and metabolism of the contrast agent in the kidney and heart, and found that the contrast agent was mildly distributed in the kidney at 1 h and apparently at 8 h (Fig. 7), and then metabolized from the body gradually. In addition, there was no signal distribution in the heart, indicating that the contrast agent had no cardiac toxicity.

## Conclusion

In this study, we acidified MWCNTs and linked them to PEG to improve their water solubility and stability. Finally, nucleic acid was attached to the surface of these nanotubes to further enhance their targeting ability and biocompatibility.

This new type of nanoultrasonic imaging material is safe and stable with a good targeting ability, showing a good clinical transformation potential (Carson et al. 2011; Kohler et al. 2005). More importantly, this new ultrasound contrast agent had a strong targeted development effect both in vivo and ex vivo. It mainly metabolized through the kidney without showing cardiopulmonary distribution. This study not only demonstrated a good ability of the new contrast agent in targeting PCa cells but provides a new approach and way of thinking in developing targeted contrast agents. In addition, since the nucleotide itself is a sequence of nucleotides, it can be inserted into anthracyclines or linked with other therapeutic genes (Justin P Dassie et al. 2009; Xu et al. 2013; Dhar et al. 2011; Kim et al. 2010). Moreover, the MWCNTs described here in had a large hydrophobic cavity and therefore could wrap many hydrophobic drugs (Sahoo et al. 2018; Kim et al. 2017). These features can be used to prepare multi-functional ultrasound contrast agents for diagnosis and treatment.

## References

[CR1] Ahmed HU, El-Shater Bosaily A, Brown LC, Gabe R, Kaplan R, Parmar MK, Collaco-Moraes Y, Ward K, Hindley RG, Freeman A, Kirkham AP, Oldroyd R, Parker C, Emberton M (2017). PROMIS study group. Diagnostic accuracy of multi-parametric MRI and TRUS biopsy in prostate cancer (PROMIS): a paired validating confirmatory study. Lancet.

[CR2] Alibolandi M, Ramezani M, Abnous K, Sadeghi F, Atyabi F, Asouri M, Ahmadi AA, Hadizadeh F (2015). In vitro and in vivo evaluation of therapy targeting epithelial-cell adhesion-molecule aptamers for non-small cell lung cancer. J Control Release.

[CR3] Baek SE, Lee KH, Park YS, Oh DK, Oh S, Kim KS, Kim DE (2014). RNA aptamer-conjugated liposome as an efficient anticancer drug delivery vehicle targeting cancer cells in vivo. J Control Release.

[CR4] Baur ADJ, Schwabe J, Rogasch J, Maxeiner A, Penzkofer T, Stephan C, Rudl M, Hamm B, Jung EM, Fischer T (2017). A direct comparison of contrast-enhanced ultrasound and dynamic contrast-enhanced magnetic resonance imaging for prostate cancer detection and prediction of aggressiveness. Eur Radiol.

[CR5] Bilimoria KY, Bentrem DJ, Feinglass JM, Stewart AK, Winchester DP, Talamonti MS, Ko CY (2008). Directing surgical quality improvement initiatives: comparison of perioperative mortality and long-term survival for cancer surgery [J]. J Clin Oncol.

[CR6] Carson AR, McTiernan CF, Lavery L, Hodnick A, Grata M, Leng X, Wang J, Chen X, Modzelewski RA, Villanueva FS (2011). Gene therapy of carcinoma using ultrasound-targeted microbubble destruction. Ultrasound Med Biol.

[CR7] Chen RJ, Zhang YG, Wang DW, Dai HJ (2001). Noncovalent sidewall functionalization of single-walled carbon nanotubes for protein immobilization. J Am Chem Soc.

[CR8] Chen Y, Yin Q, Ji X, Zhang S, Chen H, Zheng Y, Sun Y, Qu H, Wang Z, Li Y, Wang X, Zhang K, Zhang L, Shi J (2012). Manganese oxidebased multifunctionalized mesoporous silica nanoparticles for pHresponsive MRI, ultrasonography and circumvention of MDR in cancer cells. Biomaterials.

[CR9] Chu TC, Shieh F, Lavery LA, Levy M, Richards-Kortum R, Korgel BA, Ellington AD (2006). Labeling tumor cells with fluorescent nanocrystal-aptamer bioconjugates. Biosens Bioelectron.

[CR10] Dassie JP, Liu X-y, Thomas GS, Whitaker RM, Thiel KW, Stockdale KR, Meyerholz DK, McCaffrey AP, McNamara JO, Giangrande PH (2009). Systemic administration of optimized aptamer-siRNA chimeras promotes regression of PSMA-expressing tumors. Nat Biotechnol.

[CR11] Delogu LG, Vidili G, Venturelli E, Ménard-Moyon C, Zoroddu MA, Pilo G, Nicolussi P, Ligios C, Bedognetti D, Sgarrella F, Manetti R, Bianco A (2012). Functionalized multiwalled carbon nanotubes as ultrasound contrast agents. Proc Natl Acad Sci U S A.

[CR12] Dhar S, Kolishetti N, Lippard SJ, Farokhzad OC (2011). Targeted delivery of a cisplatin prodrug for safer and more effective prostate cancer therapy in vivo. Proc Natl Acad Sci U S A.

[CR13] Dougherty CA, Cai W, Hong H (2015). Applications of aptamers in targeted imaging: state of the art. Curr Top Med Chem.

[CR14] Hahn MA, Singh AK, Sharma P, Brown SC, Moudgil BM (2011). Nanoparticles as contrast agents for in-vivo bioimaging: current status and future perspectives. Anal Bioanal Chem.

[CR15] Helmstaedter L, Riemann JF (2008). Pancreatic cancer—EUS and early diagnosis [J]. Langenbeck’s Arch Surg.

[CR16] Hu H, Ni YC, Mandal SK, Montana V, Zhao B, Haddon RC (2005). Polyethyleneimine functionalized single-walled carbon nanotubes as a substrate for neuronal growth. J Phys Chem B.

[CR17] Hu H, Zhou H, Du J, Wang Z, An L, Yang H, Li F, Wua H, Yang S (2011). Biocompatiable hollow silica microspheres as novel ultrasound contrast agents for in vivo imaging. J Mater Chem.

[CR18] Kiessling F, Fokong S, Koczera P, Lederle W, Lammers T (2012). Ultrasound microbubbles for molecular diagnosis, therapy, and theranostics. J Nucl Med.

[CR19] Kim E, Jung Y, Choi H, Yang J, Suh JS, Huh YM, Kim K, Haam S (2010). Prostate cancer cell death produced by the co-delivery of Bcl-xL shRNA and doxorubicin using an aptamer-conjugated polyplex. Biomaterials.

[CR20] Kim SW, Kyung Lee Y, Yeon Lee J, Hee Hong J, Khang D (2017). PEGylated anticancer-carbon nanotubes complex targeting mitochondria of lung cancer cells. Nanotechnology.

[CR21] Kohler N, Sun C, Wang J, Zhang M (2005). Methotrexate-modified superparamagnetic nanoparticles and their intracellular uptake into human cancer cells. Langmuir.

[CR22] Lee IH, An S, Yu MK, Kwon HK, Im SH, Jon S (2011). Targeted chemoimmunotherapy using drug-loaded aptamer-dendrimer bioconjugates. J Control Release.

[CR23] Mathew A, Maekawa T, Sakthikumar D (2015). Aptamers in targeted nanotherapy. Curr Top Med Chem.

[CR24] McNamara JO, Andrechek ER, Wang Y, Viles KD, Rempel RE, Gilboa E, Sullenger BA, Giangrande PH (2006). Cell type-specific delivery of siRNAs with aptamer-siRNA chimeras. Nat Biotechnol.

[CR25] Paefgen V, Doleschel D, Kiessling F (2015). Evolution of contrast agents for ultrasound imaging and ultrasound-mediated drug delivery. Front Pharmacol.

[CR26] Pai SS, Ellington AD (2009). Using RNA aptamers and the proximity ligation assay for the detection of cell surface antigens. Methods Mol Biol.

[CR27] Ristau BT, O’Keefe DS, Bacich DJ (2014). The prostate-specifc membraneantigen: lessons and current clinical implications from 20 years of research. Urol Oncol.

[CR28] Rockey WM, Hernandez FJ, Huang SY, Cao S, Howell CA, Thomas GS, Liu XY, Lapteva N, Spencer DM, McNamara JO, Zou X, Chen SJ, Giangrande PH (2011). Rational truncation of an RNA aptamer to prostate-specific membrane antigen using computational structural modeling. Nucleic Acid Ther.

[CR29] Sahoo AK, Kanchi S, Mandal T, Dasgupta C, Maiti PK (2018). Translocation of bioactive molecules through carbon nanotubes embedded in lipid membrane. ACS Appl Mater Interfaces.

[CR30] Shakir NA, Siddiqui MM, George AK, Kongnyuy M, Ho R, Fascelli M, Merino MJ, Turkbey B, Choyke PL, Wood BJ, Pinto PA (2017). Should hypoechoic lesions on transrectal ultrasound be sampled during magnetic resonance imaging-targeted prostate biopsy?. Urology.

[CR31] Siegel RL, Miller KD, Jemal A (2016). Cancer statistics, 2016. CA Cancer J Clin.

[CR32] Ukimura O, Coleman JA, de la Taille A, Emberton M, Epstein JI, Freedland SJ, Giannarini G, Kibel AS, Montironi R, Ploussard G, Roobol MJ, Scattoni V, Jones JS (2013). Contemporary role of systematic prostate biopsies: indications, techniques, and implications for patient care. Eur Urol.

[CR33] Wang L, Li L, Guo Y, Tong H, Fan X, Ding J, Huang H (2013). Construction and in vitro/in vivo targeting of PSMA-targeted nanoscale microbubbles in prostate cancer. Prostate.

[CR34] Washington SL, Bonham M, Whitson JM, Cowan JE, Carroll PR (2012). Transrectal ultrasonography-guided biopsy does not reliably identify dominant cancer location in men with low-risk prostate cancer. BJU Int.

[CR35] Welch HG, Albertsen PC (2009). Prostate cancer diagnosis and treatment after the introduction of prostate-specific antigen screening: 1986-2005. J Natl Cancer Inst.

[CR36] Wu X, Ding B, Gao J, Wang H, Fan W, Wang X, Zhang W, Wang X, Ye L, Zhang M, Ding X, Liu J, Zhu Q, Gao S (2011). Second-generation aptamer-conjugated PSMA-targeted delivery system for prostate cancer therapy. Int J Nanomedicine.

[CR37] Wu H, Shi H, Zhang H, Wang X, Yang Y, Yu C, Hao C, Du J, Hu H, Yang S (2014). Prostate stem cell antigen antibody-conjugated multiwalled carbonnanotubes for targeted ultrasound imaging and drug delivery. Biomaterials.

[CR38] Xiao ZF, Wang L, Guo Y, Tu Z, Li L, Tong H, Xu Y, Li R, Fang K (2015). Ultrasonic nanobubbles carrying antiPSMA nanobody: construction and application in prostate cancertargeted imaging. PLoS One.

[CR39] Xiao ZF, Guo Y, Wang L, Xiong X, Zhu L, Fang K (2016). Diagnosis of prostate cancer using anti-PsMa aptamer a10-3.2-oriented lipid nanobubbles. Int J Nanomedicine.

[CR40] Xu W, Imtiaz A, Siddiqui MN, Pill S, Rosenthal K, Hasan Mukhtar B, Gonga S (2013). Aptamer-conjugated and doxorubicin-loaded unimolecular micelles for targeted therapy of prostate cancer. Biomaterials.

[CR41] Yu C, Qi Y, Xiufeng J, Shengjian Z, Hangrong C, Yuanyi Z, Yang S, Haiyun Q, Zheng W, Yaping L, Xia W, Kun Z, Linlin Z, Jianlin S (2012). Manganese oxidebased multifunctionalized mesoporous silica nanoparticles for pHresponsive MRI, ultrasonography and circumvention of MDR in Cancer Cells. Biomaterials.

